# Identifying M1 Macrophage-Related Genes Through a Co-expression Network to Construct a Four-Gene Risk-Scoring Model for Predicting Thyroid Cancer Prognosis

**DOI:** 10.3389/fgene.2020.591079

**Published:** 2020-10-29

**Authors:** Gaojian Zhuang, Yu Zeng, Qun Tang, Qian He, Guoqing Luo

**Affiliations:** ^1^The Sixth Affiliated Hospital of Guangzhou Medical University, Qingyuan People’s Hospital, Qingyuan, China; ^2^Department of Thyroid and Neck Tumor, National Clinical Research Center for Cancer, Tianjin Medical University Cancer Institute and Hospital, Key Laboratory of Cancer Prevention and Therapy, Tianjin, China; ^3^Department of Pathology, Hunan University of Chinese Medicine, Changsha, China; ^4^Department of Neurosurgery, The First Affiliated Hospital of Jinan University, Guangzhou, China

**Keywords:** M1 macrophages, CIBERSORT, weighted gene co-expression network analysis, nomogram, thyroid cancer

## Abstract

Macrophages are key innate immune cells in the tumor microenvironment that regulate primary tumor growth, vascularization, metastatic spread and response to therapies. Macrophages can polarize into two different states (M1 and M2) with distinct phenotypes and functions. To investigate the known tumoricidal effects of M1 macrophages, we obtained RNA expression profiles and clinical data from The Cancer Genome Atlas Thyroid Cancer (TCGA-THCA). The proportions of immune cells in tumor samples were assessed using CIBERSORT, and weighted gene co-expression network analysis (WGCNA) was used to identify M1 macrophage-related modules. Univariate Cox analysis and LASSO-Cox regression analysis were performed, and four genes (SPP1, DHRS3, SLC11A1, and CFB) with significant differential expression were selected through GEPIA. These four genes can be considered hub genes. The four-gene risk-scoring model may be an independent prognostic factor for THCA patients. The validation cohort and the entire cohort confirmed the results. Univariate and multivariate Cox analysis was performed to identify independent prognostic factors for THCA. Finally, a prognostic nomogram was built based on the entire cohort, and the nomogram combining the risk score and clinical prognostic factors was superior to the nomogram with individual clinical prognostic factors in predicting overall survival. Time-dependent ROC curves and DCA confirmed that the combined nomogram is useful. Gene set enrichment analysis (GSEA) was used to elucidate the potential molecular functions of the high-risk group. Our study identified four genes associated with M1 macrophages and established a prognostic nomogram that predicts overall survival for patients with THCA, which may help determine clinical treatment options for different patients.

## Introduction

Thyroid cancer (THCA) is the most common malignant endocrine tumor, and the incidence of THCA has been increasing annually worldwide in recent decades. THCA has thus become one of the fastest growing malignancies in China (Valvo and Nucera, 2019). The development of high-throughput molecular and multi-omics techniques has improved our understanding of molecular changes related to the occurrence and progression of THCA (Boufraqech and Nilubol, 2019). The development of THCA is associated with many genetic changes and molecular mechanisms in addition to DNA methylation, which has been widely studied by researchers. It is now clear that the outcome of cancer and the response to treatment are guided by the activity of multiple immune cells in the tumor (Clancy and Hovig, 2016); recent studies have confirmed that immune-related genes (IRGs) play a crucial role in the recurrence and metastasis of THCA, and reliable immune-related prognostic markers are needed to determine the prognosis of THCA patients (Iancu et al., 2020; Xue et al., 2020).

There is ample evidence to show that IRGs play an important role in the biological process of cancer (Zhu et al., 2020). Among IRGs, the most explored by researchers are those linked to tumor-associated macrophages (TAMs), which have been shown to be the most common infiltrating immune cells in the tumor microenvironment of many tumors, such as gliomas, and to play a crucial role in tumorigenesis and immunosuppression (Shan et al., 2020). By analyzing the phenotypic transformation of TAMs during tumor progression, researchers found that most M1 macrophage-related genes are highly expressed in the early stage in a variety of tumors (Ren et al., 2017), and their activation also plays a significant role in the progression of inflammatory bowel disease; in addition, these genes are major pro-inflammatory genes (Zhu et al., 2016). M1 macrophage-related genes include IL-6, IL-8, CD80, PIM1, RTP4, and SLC11A1, and studies have shown that after metformin treatment, the expression of M1-related factors in tumor cells can be enhanced or weakened, thus affecting the prognosis of the tumor (Chiang et al., 2017). A comprehensive analysis of different M1 macrophage-associated genes will help to reveal the relationship between aberrant expression of different genes and the occurrence and progression of THCA and may identify new prognostic molecules and therapeutic targets.

In recent decades, M1 macrophage-related genes have been identified in the human genome. Most of them participate in regulation of the immune microenvironment in many malignant tumors, such as head and neck squamous cell carcinomas, gastric carcinoma, and hepatocellular carcinoma (Pang et al., 2019; de Vos et al., 2020; Yamakoshi et al., 2020). While mutations in these genes are the biological basis for immune escape in various hematological malignancies, these immune escape mechanisms are characteristic of both Hodgkin’s and non-Hodgkin’s lymphomas and represent new prospects for antitumor therapies (Pizzi et al., 2016).

In this study, CIBERSORT, WGCNA, univariate Cox analysis, LASSO-Cox analysis and GEPIA were used to identify M1 macrophage-related genes in the THCA microenvironment to construct a risk-scoring model, which performed well in the validation cohort and the entire cohort.

In the entire cohort, independent prognostic factors for overall survival were determined by univariate and multivariate Cox analysis. A nomogram incorporating the risk score and clinical prognostic factors was established. Overall, our risk-scoring model and nomogram can accurately predict overall survival for patients with THCA.

## Materials and Methods

### Data Collection and Preprocessing

The Cancer Genome Atlas (TCGA^1^) is a large, free reference database for researchers that collects and collates various omics data related to cancer (Tomczak et al., 2015). From TCGA, we downloaded THCA RNA expression data from 500 tumor samples with complete clinical information (including age, gender, stage, histological type, T stage, lymph node status, and metastasis). Transcript per million (TPM) values were calculated, and the expression levels of genes were presented using the Log2 (TPM + 1) scale.

### Evaluation of Tumor-Infiltrating Immune Cells

CIBERSORT is a deconvolutional algorithm that uses the expression values of 547 marker genes to predict the proportions of 22 immune cell types from bulk tumor sample expression data through support vector regression (Newman et al., 2015). To estimate the relative proportion of 22 types of infiltrated immune cells in the tumor mass, the online analytical platform CIBERSORT^2^ was used. Data were normalized following Chen (Chen et al., 2018), and the settings for the run were 1000 permutations with quantile normalization disabled.

### Construction of Gene Co-expression Networks

The R package “WGCNA” was used to construct the co-expression network and to identify the co-expressed modules (Langfelder and Horvath, 2008). Tumor samples with a CIBERSORT *P*-values < 0.05 were retained, samples with significant outliers were excluded, and the remaining samples were considered as a discovery cohort to construct co-expression networks and identify hub genes. We used the median absolute deviation (MAD) to select 5000 highly variable genes for the WGCNA expression data. First, the gene expression data were used to obtain a Pearson correlation matrix between the genes. The Pearson correlation matrix was transformed continuously with the power adjacency function to obtain a weighted network. The power adjacency function results in an adjacency matrix, as calculated by a_ij_ = | cor(i,j)| ^β^ (cor(i,j) = Pearson’s correlation between paired genes; a_ij_ = adjacency between paired genes). A soft threshold β can improve strong correlations and weaken weak correlations. The power of β = 6 (scale-free *R*^2^ = 0.85) was selected as the soft-thresholding parameter to ensure a scale-free network, and the dynamic hybrid cut method (a bottom-up algorithm) was used to identify co-expressed gene modules.

### Identification of Clinically Significant Modules

Module eigengenes (MEs) are defined as the first principal component of a given module, and the value of MEs is that they can be considered the most representative gene expression profile for each module. The correlation between MEs and macrophages was calculated by the Pearson test to identify modules of interest. A module was considered to be significantly correlated with macrophages when *P* < 0.05. We selected the macrophage subtype of interest and the module with the highest correlation coefficient and defined it as a hub module. Gene Ontology (GO) functional enrichment analysis of the gene set contained within the hub module was performed using the R package “clusterProfiler”(Yu et al., 2012), *P*-values < 0.01 were regarded as significant.

### Identification of Hub Genes

We performed univariate Cox regression analysis on the genes in the hub module to calculate the relationship between the expression level of each gene and overall survival (OS). Next, genes with *P*-values < 0.01 were analyzed by lasso regression through the R package “glmnet,” and finally, the lasso regression screened genes with significant differences in GEPIA were used as hub genes (Friedman et al., 2010; Tang et al., 2017; Blanco et al., 2018).

### Analysis of Hub Genes and Clinical Indicators

The “beeswarm” R package was used to demonstrate the relationship between genes and clinical features (including stage, histological type, T stage, lymph node status, and metastasis), and statistical significance was analyzed by the Kruskal-Wallis test.

### Construction of the Risk-Scoring Model

The coefficients of the univariate Cox analysis of hub genes in the discovery cohort were locked in the validation cohort and entire cohort. The formula for the risk score is described below: Risk score = βgene A × expr gene A + βgene B × expr gene B + ⋅⋅ + βgene N × expr gene N, where expr was the mRNA expression of the hub genes, and β was the regression coefficient for the corresponding gene in the univariate Cox regression analysis. The optimal cut-off value was identified by the R package “survminer” to split each cohort in a proportion of 4 to 6. Patients were classified into high- and low-risk groups according to the cut-off value. Survival curves for the high- and low-risk groups were plotted using Kaplan–Meier analysis. The R package “timeROC” was used to calculate time-dependent ROC curves and AUC at 3, 5, and 8 years to assess the predictive performance of the risk-scoring model. Subsequently, we considered the samples used to construct the co-expression network as the discovery cohort and used the R package “caret” to randomly divide the THCA sample, with 60% in validation cohort A (*n* = 300) and 40% in validation cohort B (*n* = 200). The predictive value of the risk-scoring model in validation cohort A, validation cohort B and the entire cohort was then further examined.

### Building and Validating a Predictive Nomogram

To develop a nomogram, we conducted univariate and multivariate analyses using Cox regression models to identify clinical features associated with survival. We used calibration curves (by the bootstrap method with 500 resamples) to validate the nomogram, and time-dependent ROC curve and decision curve analysis (DCA) were next used to compare all and only one independent prognostic factor included in the nomogram. Compared to an all or nothing strategy, DCA identifies a range of threshold probabilities for which the magnitude of the net benefit of a model is highest and determines which of several models is optimal, and the DCA tutorial is available at http://www.decisioncurveanalysis.org along with R code (Vickers and Elkin, 2006).

### Differential Gene Expression Analysis and Gene Set Enrichment Analysis

In the entire cohort, the sample was divided into high- and low-risk groups based on the cut-off value of the risk score. The R package “limma” was used to analyze the fold changes in gene expression. Gene set enrichment analysis (GSEA), which was performed with the R package “clusterProfiler,” was used to identify the pathways in which high- and low-risk groups were significantly enriched in the reference gene set, and hallmark gene sets were selected as the reference gene set (Subramanian et al., 2005).

## Results

### Clinical Features of the Thyroid Cancer Samples

We explored the distinct potential prognostic value of different M1 macrophage-related genes for THCA. Discovery cohort was used to identify hub genes, the validation cohort and the entire cohort was used to validate the four-gene risk-scoring model, and the clinical features of THCA samples from different cohorts are shown in Supplementary Table S1.

### Proportion of Tumor-Infiltrating Immune Cells in Thyroid Cancer Samples

**FIGURE 1:**
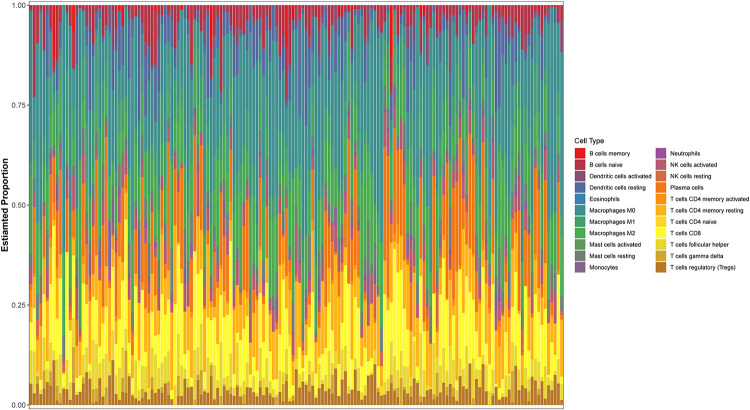
Evaluation of 22 tumor-infiltrating immune cell types.

The expression spectrum of 22 immune cells in THCA was first measured and analyzed using the CIBERSORT algorithm, and the abundance of immune cell subpopulations in the samples was evaluated. Then, we screened out the *P*-values < 0.05 samples to draw bar graphs to show the proportion of different cell subtypes in each sample (Figure 1).

### Gene Co-expression Networks of Thyroid Cancer

**FIGURE 2:**
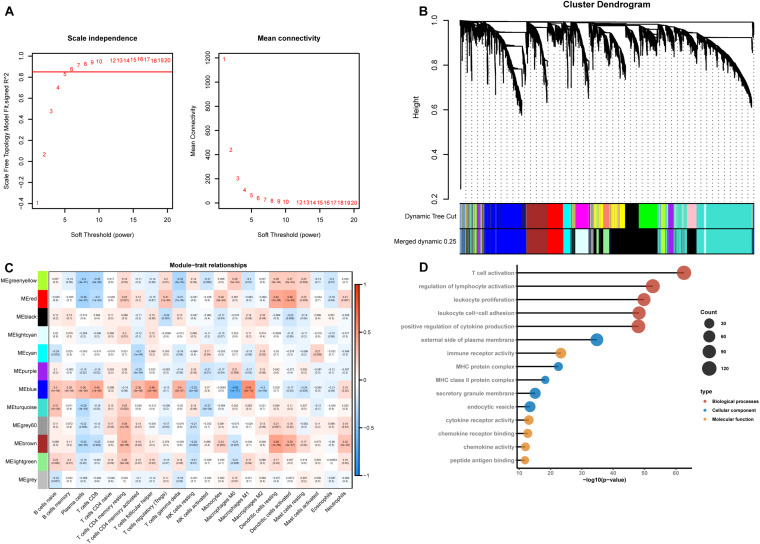
Construction of a weighted gene co-expression network. **(A)** Analysis of the scale-free network coefficient R-squared for the soft threshold (β) and the mean connectivity for the soft threshold. We hope that the value of R-squared exceeds 0.85, so the power value is 6 (β = 6). **(B)** A cluster dendrogram was built based on the dissimilarity of the topological overlap, which presents 12 network modules. **(C)** Heatmap demonstrating the correlation between module eigengenes and immune cells. **(D)** GO enrichment analysis of all genes in the hub module.

According to CIBERSORT analysis, the proportions of different cell subtypes in each sample were used as the trait data for WGCNA. Then, we used the MAD to select 5000 highly variable genes to build a co-expression network. The hclust function was used to check whether there are outliers in the sample, and 7 samples with obvious outliers were removed. Finally, 156 samples were used to construct the co-expression network. The soft threshold (power = 6) is selected as the soft threshold parameter to build the scale-free network. A total of 12 network modules were detected (Figures 2A,B). Supplementary Figure 1 shows the sample clustering plot and a heatmap of the proportions of 22 types of immune cells. The blue module is closely related to M1 macrophages (*R*^2^ = 0.54, *P* = 5e-13) and M0 macrophages (*R*^2^ = −0.62, *P* = 1e-17) (Figure 2C). The absolute value of the correlation between the other modules and macrophages was less than 0.5. We are interested in the characteristics of M1 macrophages, so the blue module was identified as a hub module. Next, we performed GO enrichment analysis of the genes in the hub module, and we showed the enrichment of the five most significant GO terms in BP, CC, and MF. The three most highly enriched terms were leukocyte proliferation, T cell activation, and regulation of the lymphocyte activation pathway (Figure 2D).

### Identification of Hub Genes and Construction of the Risk-Scoring Model

**FIGURE 3:**
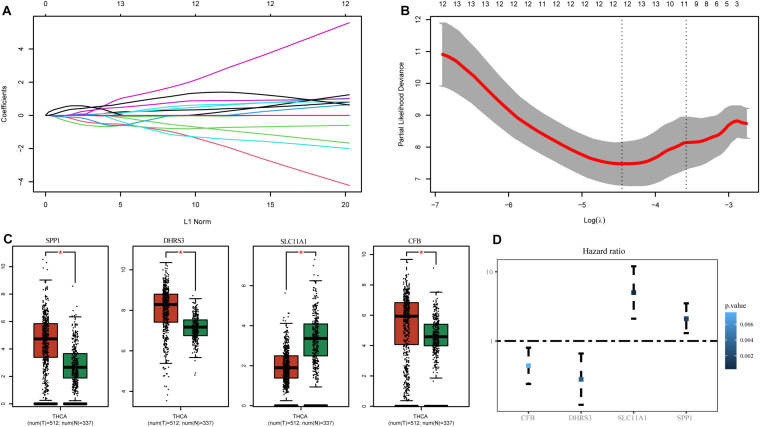
Identification of genes associated with M1 macrophages. **(A)** LASSO coefficient profiles of the prognostic-related candidate hub genes. **(B)** Relationship between partial likelihood deviance and log(λ). **(C)** Expression of the hub genes in GEPIA. **(D)** Univariate Cox analysis of the hub genes. *P-values < 0.01.

To select hub genes to construct the risk-scoring model in the TCGA training dataset, we performed univariate Cox analysis on genes within the hub module, and the results are presented in Supplementary Table S2. Univariate Cox analysis of genes *P*-values < 0.01 was performed by LASSO-Cox regression analysis to select hub genes. To determine the optimal value of the penalty parameter lambda by 10 cross-validations, lambda.min was selected as the optimal lambda value (Figures 3A,B). Based on the results of LASSO regression analysis, we filtered genes with non-zero coefficients in the LASSO model through GEPIA and selected genes with significant differential expression as hub genes (Figure 3C). Finally, the genes SPP1, DHRS3, SLC11A1, and CFB were selected as hub genes. The coefficients from univariate Cox analysis of the hub genes were used to construct the risk-scoring model (Figure 3D).

### The Relationship Between Hub Genes and Clinical Features

**FIGURE 4:**
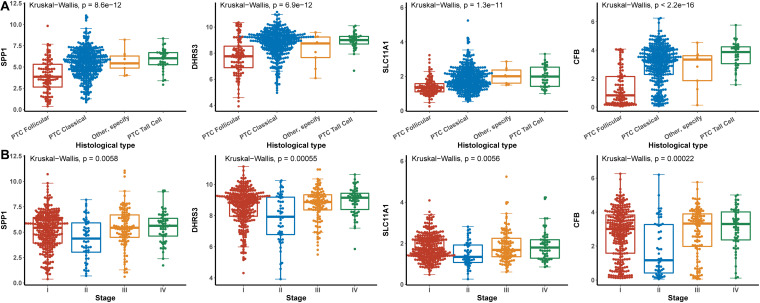
Relationship between hub genes and histological type and pathological stage. **(A)** Histological type. **(B)** Pathological stage.

**FIGURE 5:**
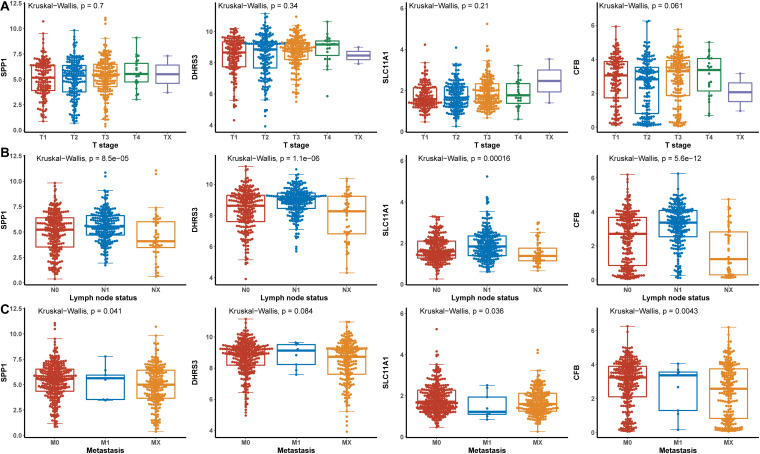
The relationship between the hub genes and TNM. **(A)** T stage. **(B)** Lymph node status. **(C)** Metastasis.

Hub genes were significantly associated with histological types and higher pathological stage and were expressed at higher levels in PTC classical cell types (Figures 4A,B). Finally, we investigated the connection between TNM and hub genes. There were no significant differences in the expression levels of all hub genes in the T stage, but the expression levels of all hub genes showed significant differences according to lymph node status and were higher in the N1 stage (Figures 5A,B). The expression levels of SPP1, SLC11A1, and CFB were differentially expressed in metastasis, although no significant differences were detected in DHRS3 (Figure 5C).

### Construction and Validation of the Risk-Scoring Model

**FIGURE 6:**
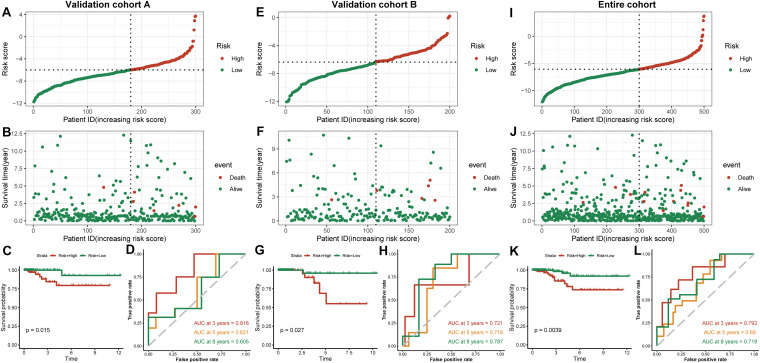
Outcomes of a four-gene risk-scoring model in each cohort. Risk score analysis, Kaplan–Meier analysis and time-dependent ROC analysis in validation cohort A **(A–D)**. Risk score analysis, Kaplan–Meier analysis and time-dependent ROC analysis in validation cohort B **(E–H)**. Risk score analysis, Kaplan–Meier analysis and time-dependent ROC analysis in the entire cohort **(I–L)**.

**FIGURE 7:**
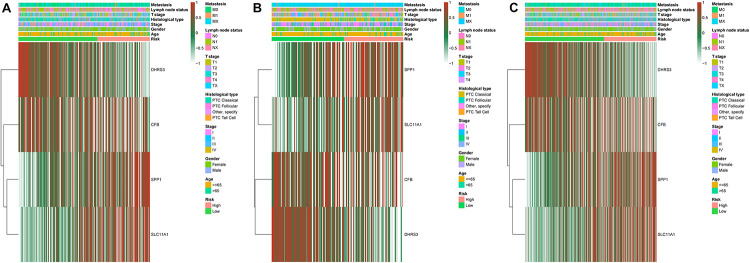
Distribution of four-gene expression profiles and clinical features of each cohort. Validation cohort A **(A)**. Validation cohort B **(B)**. Entire cohort **(C)**.

We established a risk-scoring model based on the gene expression signature of the hub genes to predict survival. The risk score formula is as follows: risk score = 2.1^∗^ expr SPP1 + 0.28^∗^ expr DHRS3 + 0.44^∗^ expr CFB + 5^∗^ expr SLC11A1. “expr” represents the expression value of the corresponding gene. To confirm the prognostic significance of the risk score. The optimal cut-off values were picked using the “survminer” package to divide the risk scores of the different cohorts into high- and low-risk groups. We validated the constructed risk-scoring model in validation cohorts A and B and the entire cohort. The population of validation cohort A was ranked according to the risk score from low to high, and we showed the survival time by the ranking (Figures 6A,B). We found that the survival curves were significantly different between the high- and low-risk groups, while the low-risk group had a better survival period (Figure 6C). The risk-scoring model of validation cohort A showed that the area under the curve (AUC) values of the time-dependent ROC curve of the 3-, 5-, and 8-year OS were 0.816, 0.621, and 0.605, respectively (Figure 6D). The population of validation cohort B was ranked according to the risk score from low to high, and we showed the survival time by the ranking (Figures 6E,F). We found that the survival curves were significantly different between the high- and low-risk groups, while the low-risk group had a better survival period (Figure 6G). The risk-scoring model of validation cohort B showed that the AUC values of the time-dependent ROC curve of the 3-, 5-, and 8-year OS were 0.721, 0.716, and 0.787, respectively (Figure 6H). The population of the entire cohort was ranked according to the risk score from low to high, and we showed the survival time by the ranking (Figures 6I,J). We found that the survival curves were significantly different between the high- and low-risk groups, while the low-risk group had a better survival period (Figure 6K). The risk-scoring model of entire cohort showed that the AUC values of the time-dependent ROC curve of the 3-, 5-, and 8-year OS were 0.793, 0.69, and 0.719, respectively (Figure 6L). The relationships between hub gene heat maps and clinical features in validation cohort A, validation cohort B and the entire cohort are shown in Figure 7.

### Establishment of a Nomogram for Overall Survival Prediction in THCA

**FIGURE 8:**
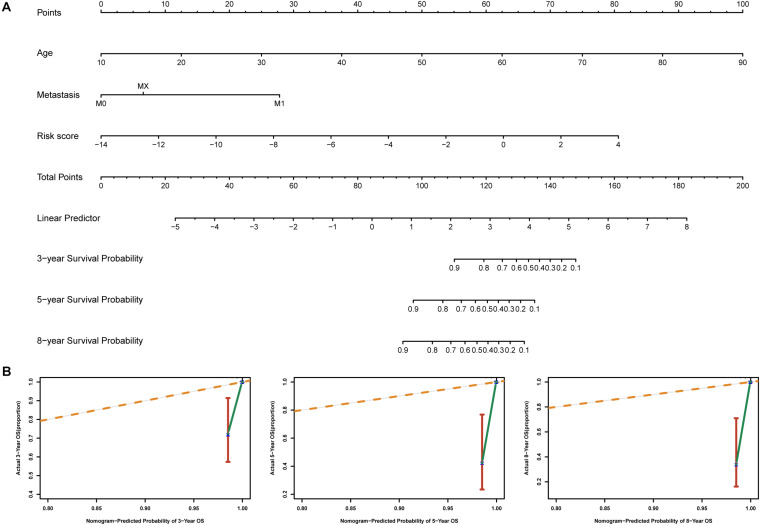
Nomogram for predicting overall survival in THCA patients. **(A)** Construction of the nomogram was based on age, metastasis and risk score in the entire cohort. **(B)** The calibration plot for internal validation of the nomogram.

**FIGURE 9:**
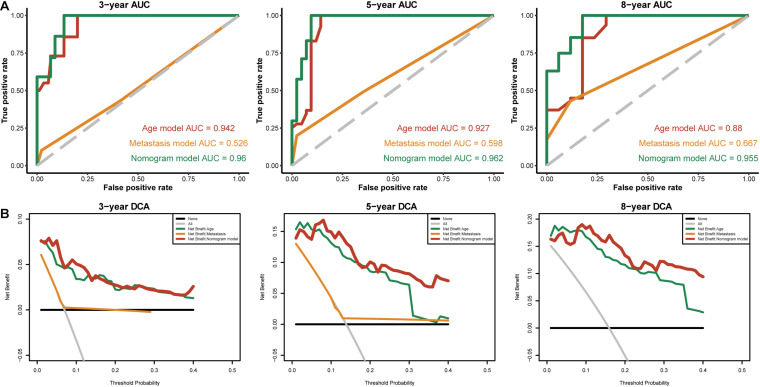
The time-dependent ROC and DCA curves of the nomogram. **(A)** The time-dependent ROC curves of the different nomograms compared for 3-, 5-, and 8-year overall survival in the entire cohort. **(B)** The DCA curves of the different nomograms compared for 3-, 5-, and 8-year overall survival in the entire cohort. The 8-year DCA curves of metastasis are not shown, as the calculated net benefit were all smaller than calculated with the none assumption.

To identify independent prognostic factors, we performed univariate and multivariate Cox analyses in the entire cohort, which showed that age, metastasis and risk score were independent prognostic factors for OS in THCA (Supplementary Table S3). We developed a nomogram that can predict OS for THCA at 3, 5, and 8 years using age, metastasis and risk score (Figure 8A). Calibration charts showed that the nomogram model may underestimate or overestimate mortality (Figure 8B). Compared with the nomogram including only age or metastasis, the nomogram model showed the largest AUC for 3-year OS, 5-year OS and 8-year OS (Figure 9A). DCA demonstrated that the nomogram model had the best net benefits for 3-year, 5-year, and 8-year OS (Figure 9B). In conclusion, the above results suggest that the nomogram that combines the risk score and clinical features performs well in predicting both short-term survival (3 years) and long-term survival (5 and 8 years) when compared to the nomogram constructed using a single prognostic factor. Nomograms combining clinical features and risk score may facilitate patient counseling, decision-making, and clinical management.

### Gene Set Enrichment Analysis of High- and Low-Risk Score Groups

**FIGURE 10:**
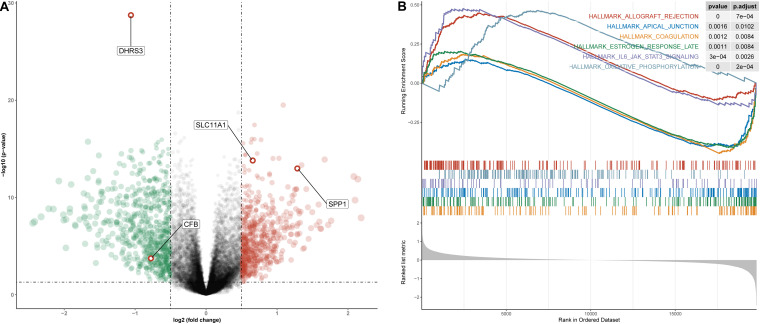
Differential gene expression analysis and GSEA for high- and low-risk groups in the entire cohort. **(A)** Volcano plot for differentially expressed genes. Red dots represent genes whose expression is upregulated, green dots represent genes whose expression is downregulated, and red circles represent hub genes. **(B)** The three pathways with the smallest NES and the three pathways with the largest NES are displayed.

To understand how the high- and low-risk score groups affect THCA survival, we used the optimal cut-off value of the risk score to divide the entire cohort into high- and low-risk groups for GSEA. The volcano plot shows the results of differential gene expression analysis (Figure 10A). We show the three pathways with the maximum normalized enrichment score (NES) values and the three pathways with the minimum NES (Figure 10B). According to the results, the high-risk score group may be associated with oxidative phosphorylation of proteins, transplant rejection, and the IL-6/JAK/STAT3 pathway, and the low-risk group may be associated with components of the blood coagulation system, late responses to estrogen and the apical junction complex (Supplementary Table S4).

## Discussion

M1 macrophage-related genes are not only closely associated with the development and promotion of inflammation but are also involved in the infiltration and metastasis of a variety of malignant tumor cells. Although some members of the M1 macrophage-related gene family have been confirmed to play critical roles in THCA, the different functions of M1 macrophage-related gene family members in THCA are still unclear. In this study, the expression and prognostic values of different M1 macrophage-related genes in THCA were analyzed, and the combined prognostic gene signature may have a better predictive effect than a single biomarker.

There has been an extraordinary amount of research showing that the increased expression of several M1 macrophage-related genes often predicts a poor prognosis. In this study, we established a novel four-gene signature (including SPP1, DHRS3, CFB, and SLC11A1) for THCA prognosis prediction. The risk-scoring model not only has good predictive performance in validation cohort A, but also has good predictive performance in validation cohort B. The four-gene risk-scoring model was an independent prognostic factor for THCA, and the survival rate of patients in the high-risk group is significantly lower than that in the low-risk group. The AUC values of the risk-scoring model in each cohort (validation cohort A, validation cohort B, and the entire cohort) and the nomogram combining the risk-scoring model and clinical prognostic factors performed well in predicting short-term survival (3 year) and long-term survival (5- and 8-year) for patients with THCA. Furthermore, GSEA revealed several significantly enriched pathways. The high-risk group was associated with oxidative phosphorylation of proteins and the IL-6/JAK/STAT3 pathway, while the low-risk group was associated with components of the blood coagulation system and late responses to estrogen, which might help explain the underlying molecular mechanisms that the high- and low-risk groups have different OS.

Secreted Phosphoprotein 1 (SPP1), also known as osteopontin, is one of the molecules in the PI3K/AKT/mTOR pathway, and it has been reported as an oncogene in many cancers (Yang et al., 2020; Li et al., 2018; Guo et al., 2020). It is also a cytokine that can upregulate the expression of interferon and interleukin-12 (Wu Q. et al., 2019). Studies have found that SPP1 can promote the proliferation, migration and invasion of malignant tumor cells and inhibit cell apoptosis, leading to poor prognosis in certain tumors (Liu et al., 2020). However, the function and mechanism of abnormal SPP1 in THCA patients are still unclear. Our study found that SPP1 was significantly overexpressed in tumor samples, and its univariate Cox analysis in the discovery cohort showed it to be a risk factor for patients with THCA.

Dehydrogenase/reductase member 3 (DHRS3) is involved in tumor suppression pathways and is constitutively expressed in breast cancer cell lines (Zhang et al., 2019). DHRS3, as a retinaldehyde reductase, can maintain embryonic development, cell differentiation and apoptosis by maintaining the cell supply of retinol and its metabolites (Kirschner et al., 2010; Wu L. et al., 2019). It has been confirmed that DHRS3 is highly expressed in PTC, as confirmed by our study, and it may become a potential target for PTC therapy (Oler et al., 2008).

Solute Carrier Family 11 member 1 (SLC11A1) is a phagosomal membrane protein that is expressed in monocytes, and monocytes are the circulating precursors of macrophages and dendritic cells (DCs) (Bauler et al., 2017). SLC11A1, as a pro-inflammatory factor, is closely related to the occurrence and progression of many inflammatory diseases (Xu and Yang, 2020). In addition, it is also associated with susceptibility to many infectious diseases (Braliou et al., 2019). We show that SLC11A1 is expressed at low levels in tumor samples, and it has been suggested that transcriptional repression of SLC11A1 leads to cell proliferation and survival if unchecked, which could result in cancer and autoimmunity (Awomoyi, 2007).

Complement Factor B (CFB) is one of the factors of the complement system, and after interacting with the tumor cell lines MDA-MB231, CFB can show significant upregulation (Oliveira-Ferrer et al., 2020). Studies have shown that CFB may be a new tumor marker for the diagnosis of pancreatic cancer. An increased level of CFB has very high sensitivity and specificity for the diagnosis of pancreatic cancer, and its false-positive rate is lower than that of the common tumor marker CA19-9 (Kim et al., 2019). The complement system is a powerful system with a wide range of biological functions, and the activation of its significant components can control tumor growth (Gadwa and Karam, 2020). We designed the nomogram to combine four gene signatures and clinical features. Its visual scoring system is easy to understand and facilitates individualized therapies and clinical decision-making. Patients with high risk scores should be treated aggressively, while patients with low risk scores should avoid additional treatments that may lead to unnecessary toxicity.

To our knowledge, the prognostic model and nomogram associated with these four-gene signature have not been reported previously and they could be a useful prognostic method for THCA. However, our study has some limitations. First, the clinical information on THCA that we downloaded from TCGA was limited and incomplete. Second, detailed information about family history, tumor size, vascular tumor invasion, extent of tumor resection and regional factors were not included in the nomogram. Last, nomograms are based on a retrospective study design, and multicentre, large-scale prospective clinical trials are required to further validate the model.

## Conclusion

This study identified genes associated with M1 macrophages through co-expression networks and constructed a risk-scoring model based on four genes and a nomogram incorporating the risk-scoring model and clinical features to predict patient prognosis and guide clinical decision-making. However, further research is needed to explore the biological role of these four genes in THCA and to incorporate more detailed clinical data to construct a broadly applicable nomogram. We hope the present study will provide powerful evidence for the future development of individualized genomic treatment in THCA.

## Data Availability Statement

Publicly available datasets were analyzed in this study. This data can be found here: https://portal.gdc.cancer.gov/.

## Author Contributions

GZ and YZ analyzed the data and wrote the manuscript. GZ and GL designed the study. GL, QT, and QH assisted with writing the manuscript. All authors read and approved the final manuscript.

## Conflict of Interest

The authors declare that the research was conducted in the absence of any commercial or financial relationships that could be construed as a potential conflict of interest.

## Footnotes

1 http://cancergenome.nih.gov/

2 https://cibersort.stanford.edu/

## Supplementary Material

Supplementary Figure S1 Sample clustering (Discovery cohort). Sample dendrogram and trait heatmap. On the heatmap, red represents a higher percentage of immune cell infiltration.

Supplementary Table S1 Clinical features of the thyroid cancer samples in each cohort.

*P*_*A*_: Comparison of validation cohort A and the discovery cohort. *P*_*B*_: Comparison of validation cohort A and the discovery cohort.

Supplementary Table S2 Univariate cox analysis within the hub module.

Supplementary Table S3 Univariate and multivariate Cox analyses of the clinical features in the entire cohort.

Supplementary Table S4 GSEA results.

## Abbreviations

THCA: thyroid cancer
PTC: papillary thyroid cancer
TME: tumor microenvironment
TAMs: tumor-associated macrophages
MHC: major histocompatibility complex
TCGA: the cancer genome atlas
WGCNA: weighted gene co-expression network analysis
GO: gene ontology
GSEA: gene set enrichment analysis
AUC: the area under the curve
DCA: decision curve analysis.
