# Supplementary material for: Identifying M1 Macrophage-Related Genes Through a Co-expression Network to Construct a Four-Gene Risk-Scoring Model for Predicting Thyroid Cancer Prognosis
Source: Front Genet. 2020 Oct 29;11:591079. doi: 10.3389/fgene.2020.591079 (PMC7658400; doi:10.3389/fgene.2020.591079)
Supplement: Supplementary Figure S1 — Sample clustering (Discovery cohort). Sample dendrogram and trait heatmap. On the heatmap, red represents a higher percentage of immune cell infiltration. [file Image_1.PDF]

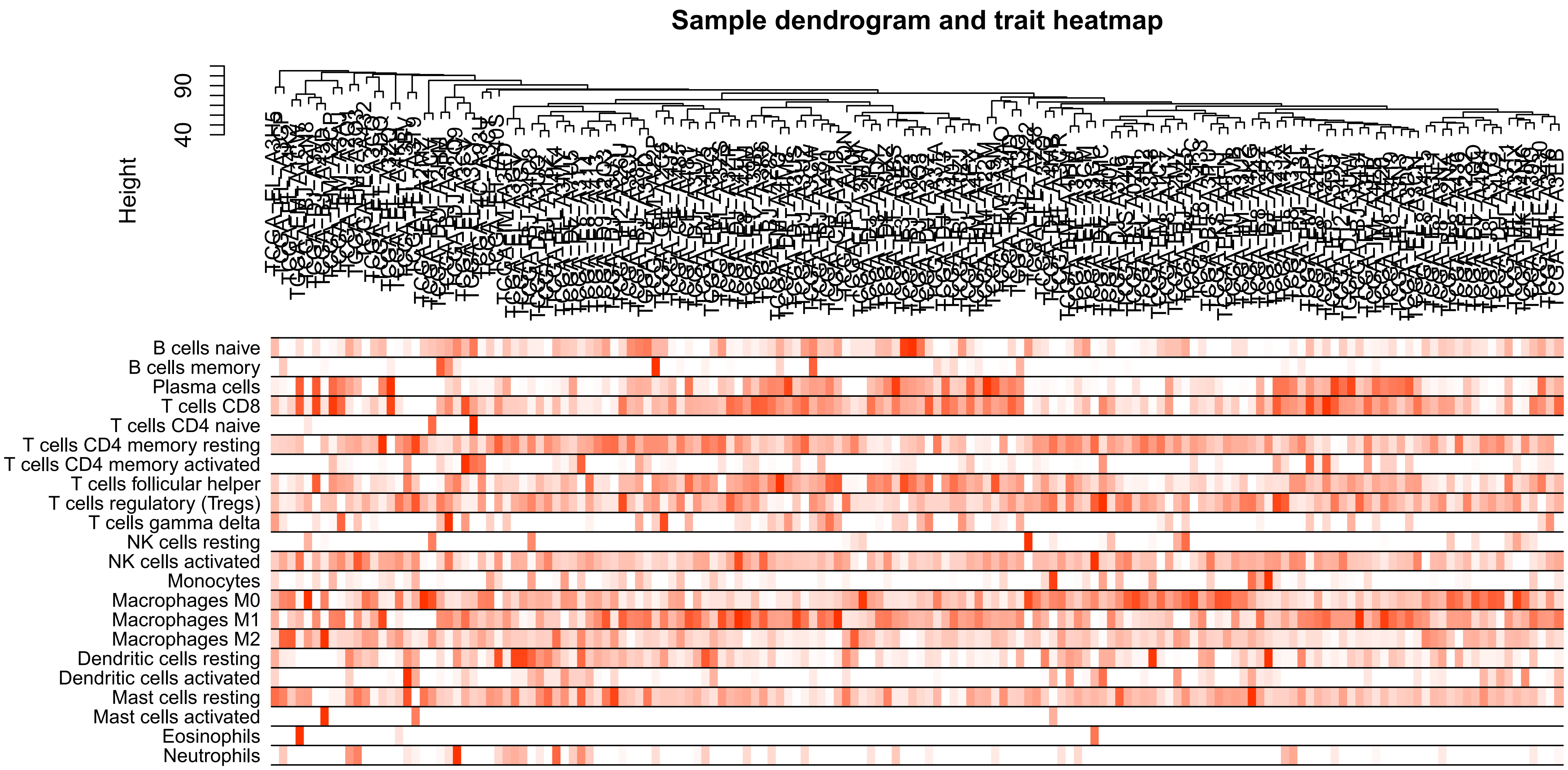

Supplementary Figure 1. Sample clustering (Discovery cohort). Sample dendrogram and trait heatmap. On the heatmap, red represents a higher percentage of immune cell infiltration.
