# Supplementary material for: Identifying M1 Macrophage-Related Genes Through a Co-expression Network to Construct a Four-Gene Risk-Scoring Model for Predicting Thyroid Cancer Prognosis
Source: Front Genet. 2020 Oct 29;11:591079. doi: 10.3389/fgene.2020.591079 (PMC7658400; doi:10.3389/fgene.2020.591079)
Supplement: Supplementary Table S1 — Clinical features of the thyroid cancer samples in each cohort. [file Table_1.docx]

**Supplementary Table S1**

Clinical features of the thyroid cancer samples in each cohort

| **Variables** | **Discovery cohort** | **Validation cohort A** | ***P_A_*** | **Validation cohort B** | ***P_B_*** | **Entire cohort** |
| --- | --- | --- | --- | --- | --- | --- |
|  | **(n=156)** | **(n=300)** |  | **(n=200)** |  | **(n=500)** |
| **Age (mean (SD))** | 49.16 (16.98) | 48.69 (15.93) | 0.772 | 47.80 (15.78) | 0.436 | 48.34 (15.86) |
| **Gender = Male (%)** | 41 (26.3) | 89 (29.7) | 0.516 | 46 (23.0) | 0.555 | 135 (27.0) |
| **Stage (%)** |  |  | 0.115 |  | 0.452 |  |
| I | 91 (58.3) | 166 (55.3) |  | 115 (57.5) |  | 281 (56.2) |
| II | 8 (5.1) | 34 (11.3) |  | 18 (9.0) |  | 52 (10.4) |
| III | 37 (23.7) | 73 (24.3) |  | 39 (19.5) |  | 112 (22.4) |
| IV | 20 (12.8) | 27 (9.0) |  | 28 (14.0) |  | 55 (11.0) |
| **Histological type (%)** |  |  | 0.004 |  | <0.001 |  |
| PTC Classical | 132 (84.6) | 219 (73.0) |  | 136 (68.0) |  | 355 (71.0) |
| PTC Follicular | 9 (5.8) | 54 (18.0) |  | 46 (23.0) |  | 100 (20.0) |
| Other, specify | 2 (1.3) | 6 (2.0) |  | 3 (1.5) |  | 9 (1.8) |
| PTC Tall Cell | 13 (8.3) | 21 (7.0) |  | 15 (7.5) |  | 36 (7.2) |
| **T stage (%)** |  |  | 0.786 |  | 0.17 |  |
| T1 | 51 (32.7) | 83 (27.7) |  | 58 (29.0) |  | 141 (28.2) |
| T2 | 41 (26.3) | 91 (30.3) |  | 73 (36.5) |  | 164 (32.8) |
| T3 | 56 (35.9) | 113 (37.7) |  | 57 (28.5) |  | 170 (34.0) |
| T4 | 7 (4.5) | 11 (3.7) |  | 12 (6.0) |  | 23 (4.6) |
| TX | 1 (0.6) | 2 (0.7) |  | 0 (0.0) |  | 2 (0.4) |
| **Lymph node status (%)** |  |  | 0.089 |  | 0.028 |  |
| N0 | 65 (41.7) | 142 (47.3) |  | 86 (43.0) |  | 228 (45.6) |
| N1 | 84 (53.8) | 133 (44.3) |  | 90 (45.0) |  | 223 (44.6) |
| NX | 7 (4.5) | 25 (8.3) |  | 24 (12.0) |  | 49 (9.8) |
| **Metastasis (%)** |  |  | 0.76 |  | 0.013 |  |
| M0 | 100 (64.1) | 184 (61.3) |  | 97 (48.5) |  | 281 (56.2) |
| M1 | 2 (1.3) | 6 (2.0) |  | 3 (1.5) |  | 9 (1.8) |
| MX | 54 (34.6) | 110 (36.7) |  | 100 (50.0) |  | 210 (42.0) |

*P_A_*: Comparison of validation cohort A and the discovery cohort. *P_B_*: Comparison of validation cohort A and the discovery cohort.
