# Supplementary material for: Identifying M1 Macrophage-Related Genes Through a Co-expression Network to Construct a Four-Gene Risk-Scoring Model for Predicting Thyroid Cancer Prognosis
Source: Front Genet. 2020 Oct 29;11:591079. doi: 10.3389/fgene.2020.591079 (PMC7658400; doi:10.3389/fgene.2020.591079)
Supplement: Supplementary Table S3 — Univariate and multivariate Cox analyses of the clinical features in the entire cohort. [file Table_3.docx]

**Supplementary Table S3**

Univariate and multivariate Cox analyses of the clinical features in the entire cohort.

| **Variables** | **Univariate Cox analysis** | |  | **Multivariate Cox analysis** | |
| --- | --- | --- | --- | --- | --- |
|  | **HR (95% CI for HR)** | **p.value** |  | **HR (95% CI for HR)** | **p.value** |
| **Age** | 1.14 (1.08-1.19) | <0.001 |  | -0.172 (1.04-1.18) | 0.002 |
| **Gender** | 2.01 (0.673-6.03) | 0.211 |  | -0.172 (0.195-3.63) | 0.817 |
| **Stage** | 9.87 (2.74-35.5) | <0.001 |  | -0.172 (0.254-44.1) | 0.359 |
| **Histological type** | 3.68e-08 (0-Inf) | 0.998 |  | -0.172 (0-Inf) | 0.999 |
| **T stage** | 2.46 (0.771-7.87) | 0.128 |  | -0.172 (0.0517-2.14) | 0.246 |
| **Lymph node status** | 1.42 (0.492-4.1) | 0.518 |  | -0.172 (0.0592-1.59) | 0.16 |
| **Metastasis** | 5.41 (1.2-24.3) | 0.028 |  | -0.172 (1.8-85.4) | 0.011 |
| **Risk score** | 1.64 (1.36-1.98) | <0.001 |  | -0.172 (1.19-2.09) | 0.002 |
